# Electrophysiological Properties of Medium Spiny Neuron Subtypes in the Caudate-Putamen of Prepubertal Male and Female *Drd1a*-tdTomato Line 6 BAC Transgenic Mice

**DOI:** 10.1523/ENEURO.0016-19.2019

**Published:** 2019-03-13

**Authors:** Jaime A. Willett, Jinyan Cao, David M. Dorris, Ashlyn G. Johnson, Laura A. Ginnari, John Meitzen

**Affiliations:** 1 Department of Biological Sciences; 2 W.M. Keck Center for Behavioral Biology; 3 Graduate Program in Physiology, North Carolina State University, Raleigh, North Carolina 27695; 4 Neuroscience Graduate Program, Emory University, Atlanta, Georgia 30322; 5 Center for Human Health and the Environment; 6 Comparative Medicine Institute, North Carolina State University, Raleigh, North Carolina 27695

**Keywords:** caudate putamen, electrophysiology, intrinsic excitability, medium spiny neurons, rodent, sex differences

## Abstract

The caudate-putamen is a striatal brain region essential for sensorimotor behaviors, habit learning, and other cognitive and premotor functions. The output and predominant neuron of the caudate-putamen is the medium spiny neuron (MSN). MSNs present discrete cellular subtypes that show differences in neurochemistry, dopamine receptor expression, efferent targets, gene expression, functional roles, and most importantly for this study, electrophysiological properties. MSN subtypes include the striatonigral and the striatopallidal groups. Most studies identify the striatopallidal MSN subtype as being more excitable than the striatonigral MSN subtype. However, there is some divergence between studies regarding the exact differences in electrophysiological properties. Furthermore, MSN subtype electrophysiological properties have not been reported disaggregated by biological sex. We addressed these questions using prepubertal male and female Drd1a-tdTomato line 6 BAC transgenic mice, an important transgenic line that has not yet received extensive electrophysiological analysis. We made acute caudate-putamen brain slices and assessed a robust battery of 16 relevant electrophysiological properties using whole-cell patch-clamp recording, including intrinsic membrane, action potential, and miniature EPSC (mEPSC) properties. We found that: (1) MSN subtypes exhibited multiple differential electrophysiological properties in both sexes, including rheobase, action potential threshold and width, input resistance in both the linear and rectified ranges, and mEPSC amplitude; (2) select electrophysiological properties showed interactions between MSN subtype and sex. These findings provide a comprehensive evaluation of mouse caudate-putamen MSN subtype electrophysiological properties across females and males, both confirming and extending previous studies.

## Significance Statement

The findings presented here provide a comprehensive evaluation of the electrophysiological properties of caudate-putamen medium spiny neuron (MSN) subtypes, both in terms of electrophysiological metrics and animal sex. These data selectively confirm, diverge from, and extend the findings of previous studies, providing a firm foundation on which to pursue future studies of caudate-putamen MSNs.

## Introduction

The most abundant neuron type in the mammalian caudate-putamen is the medium spiny neuron (MSN), also called the spiny projection neuron (Graveland and Difiglia, 1985; Gerfen and Surmeier, 2011). The MSN is the output neuron of the caudate-putamen and other striatal brain regions, and is implicated in a wide range of cognitive and sensorimotor behaviors and relevant striatal disorders (Kreitzer and Malenka, 2008; Koob and Volkow, 2010; Maia and Frank, 2011). To regulate these behaviors, MSNs integrate glutamatergic, dopaminergic, GABAergic, cholinergic, estrogenic, and other inputs to influence both internal and external targets. MSNs are phenotypically diverse, encompassing at least two different subtypes, which differ in neurochemistry, dopamine receptor expression, efferent targets, gene expression, functional roles, and electrophysiological properties (Gerfen et al., 1990; Cepeda et al., 2008; Gertler et al., 2008; Shuen et al., 2008; Ade et al., 2011; Kramer et al., 2011; Kreitzer and Berke, 2011; Chan et al., 2012; Kravitz et al., 2012; Ma et al., 2012, 2013; Planert et al., 2013; Fieblinger et al., 2014; Friend and Kravitz, 2014; Keeler et al., 2014; Gokce et al., 2016; Schier et al., 2017; Sebel et al., 2017; Ho et al., 2018).

These two MSN subtypes include the striatonigral and the striatopallidal. Striatonigral MSNs express D1 dopamine receptors, which are the product of the gene *Drd1a*, and contain the neuropeptides substance P and dynorphin. Striatopallidal MSNs express D2 dopamine receptors, which are the product of the gene *Drd2*, and contain the neuropeptide enkephalin. Previous studies exploring caudate-putamen MSN subtype-specific electrophysiological properties have generally identified the Drd2-expressing subtype as being more excitable compared with the Drd1a-expressing subtype. However, there is some divergence between studies regarding the exact differences in electrophysiological properties (Table 1), and few studies have comprehensively evaluated a wide variety of cellular electrophysiological properties in individual MSNs of identified subtypes. Furthermore, all previous studies of caudate-putamen MSN subtypes have been performed in rats or mice of either solely male or unreported sex, typical of the majority of neuroscience preclinical studies (Beery and Zucker, 2011; Shansky and Woolley, 2016; Will et al., 2017). This is problematic given that striatal-mediated behaviors and disorders exhibit sex differences and/or sex steroid hormone sensitivity in phenotype and/or incidence (Calhoun, 1962; Eckel et al., 2000; Zurkovsky et al., 2007; Hosseini-Kamkar and Morton, 2014; Yoest et al., 2018), and that striatal region and developmental stage-influenced sex differences exist in MSN electrophysiological properties, at least in rats (Arnauld et al., 1981; Tansey et al., 1983; Mermelstein et al., 1996; Wissman et al., 2011; Dorris et al., 2015; Tozzi et al., 2015; Cao et al., 2016, 2018; Willett et al., 2016; Proaño et al., 2018).

**Table 1.** Drd1a and Drd2 caudate-putamen medium spiny neuron properties compared across studies

**Property**	Kreitzer and Malenka, 2007^a^	Gertler et al., 2008	Cepeda et al., 2008	Ade et al., 2008^c^	Chan et al., 2012	Planert et al., 2013	Planert et al., 2013	Goodliffe et al., 2018	**Current study, 2019**
Animal	Mice	Mice	Mice	Mice	Mice	Mice	Rats	Mice	Mice
MSN subtype identification	M4- or D2-eGFP BAC transgenic mice	D1 and D2 receptor-eGFP BAC transgenic mice on an FVB background	D1 and D2 receptor-eGFP BAC transgenic mice	D1 and D2 receptor-eGFP BAC transgenic mice on a C57BL/6J background	D1 and D2 receptor-eGFP BAC transgenic mice on either a FVB/NJ or C57BL/6J background	D1 receptor-eGFP BAC transgenic mice	Retrograde labeling of striatonigral MSNs	D1 and D2 receptor-eGFP BAC transgenic mice on a C57BL/6J background	B6 Cg-Tg (Drd1a-tdTomato) 6 Calak/J hemizygous mice ona C57BL/6J background
Animal age	P20–P25	P17–P70	P39.7 + 1.6	P16–P25	P21–P35	P15, P21–P32	P14–P19	∼P365	P17–P22
Animal sex	Not reported	Not reported	Not reported	Male and female data pooled regardless of sex	Male	Not reported	Not reported	Male and Female data pooled regardless of sex	Male and Female data analyzed by sex
Resting membrane potential	D1 = D2	D1<D2	—	D1 = D2	—	D1 = D2	D1 = D2	D1 = D2	D1 = D2
Rheobase	—	D1>D2	—	—	—	D1>D2	D1>D2	D1>D2	D1>D2
AP threshold	—	D1 = D2	D1>D2	—	—	D1 = D2	D1 = D2	D1>D2	D1>D2
AP amplitude	—	—	D1 = D2	—	—	—	—	D1 = D2	D1 = D2
AP amplitude change from first to second AP	—	—	—	—	—	D1 = D2	D1>D2	—	—
AP width	—	—	D1 = D2	—	—	D1 = D2	D1 = D2	—	D1>D2
AHP peak	—	—	D1 = D2	—	—	—	—	—	D1 = D2
AHP time to Peak	—	—	—	—	—	—	—	—	D1 = D2
Frequency of evoked action potentials/FI slope	D1<D2	D1<D2	—	D1<D2	D1<D2	D1 = D2	D1 = D2	—	D1<D2
Linear range input resistance	D1 = D2	D1<D2	D1 = D2	D1 = D2	—	D1 = D2	D1<D2	D1 = D2	D1<D2
Rectified range input resistance	—	—	—	—	—	D1 = D2	D1 = D2	—	D1 = D2
Inward rectification, %	—	—	—	—	—	—	—	—	D1 = D2
Time constant of the membrane	—	D1>D2	D1 = D2	—	—	D1 = D2	D1<D2	D1<D2	D1 = D2
Capacitance	—	D1>D2	D1 = D2	—	—	—	—	—	D1 = D2
sEPSC frequency	—	—	D1<D2	—	—	—	—	—	—
sEPSC amplitude	—	—	D1 = D2	—	—	—	—	—	—
sEPSC kinetics	—	—	D1 = D2	—	—	—	—	—	—
mEPSC frequency	D1<D2	—	D1<D2	—	—	—	—	D1 = D2	D1 = D2
mEPSC amplitude	D1 = D2	—	D1 = D2	—	—	—	**—**	D1 = D2	D1<D2
mEPSC decay	D1 = D2	—	D1 = D2	—	—	—	—	—	D1 = D2
mEPSC rise time	—	—	D1<D2	—	—	—	—	—	D1 = D2
s/mIPSC frequency	—	—	—	D1 = D2	—	—	—	—	—
s/mIPSC amplitude	—	—	—	D1 = D2	—	—	—	—	—
s/mIPSC decay	—	—	—	D1 = D2	—	—	—	—	—
s/mIPSC rise time	—	—	—	D1 = D2	—	—	—	—	—
Probability of occurrence of spontaneous membrane depolarization after GABA_A_ blockade	—	—	D1<D2	—	—	—	—	—	—
Paired-pulse ratio	D1>D2	—	D1<D2	—	—	—	—	—	—
AMPA-induced current amplitude	—	—	D1>D2	—	—	—	—	—	—
NMDA/AMPA ratio	D1<D2	—	—	—	—	—	—	—	—
Endocannabinoid-mediated LTD	D1<D2	—	—	—	—	—	—	—	—
Tonic GABA_A_ current and sensitivity to GABA_A_ current	—	—	D1<D2	D1<D2	—	—	—	—	—

Only caudate-putamen MSN subtype electrophysiology studies in acute brain slice preparation experiments independent of variables such as dopamine depletion and psychostimulant exposure are included. This criteria a priori excludes studies that analyzed MSN subtype electrophysiological properties but did not directly compare D1 and D2 subtype groups (Day et al., 2006; Ade et al., 2011), or were performed in regions such as the nucleus accumbens (Ma et al., 2012; Cao et al., 2018). —, Did not measure; AP, action potential; AHP, afterhyperpolarization; FI, Frequency of evoked spikes to injected depolarization current; LTD, long-term depression.

*^a^*The use of M4 eGFP labeling as equivalent to the D1 MSN subtype has been cautioned (Cepeda et al., 2008).

*^b^*This finding significant in some but not all analyses within this study.

*^c^*A number of studies from Vicini and colleagues have investigated GABA conductance between MSN subtypes; here we feature the initial report.

*^d^*Planert et al. (2013) assessed rheobase using multiple analyses. The conclusion of all analyses was similar and is thus condensed here.

To address these gaps in knowledge, we used female and male B6 *Cg-Tg (Drd1a-*tdTomato) line 6 Calak/J hemizygous mice, a bacterial artificial chromosome (BAC) transgenic mouse line initially developed in the laboratory of Dr. Nicole Calakos at Duke University (Ade et al., 2011). This mouse line and many others are widely used for experiments targeting neuronal subtypes (Valjent et al., 2009; Ting and Feng, 2014). An advantage of this particular BAC transgenic line is that it expresses a sensitive and specific fluorescent reporter for the Drd1a-expressing MSN subtype, enabling accurate identification of MSN subtypes within a single mouse. Other advantages of this mouse line compared with other candidates are that this line exhibits normal caudate-putamen-mediated behaviors and does not appear to show obvious cellular or physical confounds (Ade et al., 2011; Enoksson et al., 2012; Thibault et al., 2013). We made acute brain slices of male and female mouse caudate-putamen and then recorded individual MSN subtypes using whole-cell patch-clamp. We analyzed a comprehensive battery of caudate-putamen MSN subtype electrophysiological attributes to test the hypothesis that MSN electrophysiological properties differs by subtype across both males and females, including action potential, excitability, passive membrane and input resistance properties, and miniature EPSCs (mEPSCs).

## Materials

### Animals

Male B6 *Cg-Tg (Drd1a-*tdTomato) line 6 Calak/J mice and female C57BL/6 background mice were purchased from The Jackson Laboratory (JAX stock #16204). During the first week after arrival mice were individually housed. After the first week mice were housed in male and female pairs to enable breeding of hemizygous offspring. Offspring aged postnatal day (P)17–P22 from F1 litters were used in experiments (*n* = 25) and were matched between experimental groups (10 Drd1a male mice: P19.4 ± 0.2; 7 Drd1a female mice: P21.0 ± 0.2; 4 Drd2 male mice: P19.8 ± 0.5; 3 Drd2 female mice: P20.0 ± 0.7; *p* > 0.05). Approximately three neurons were recorded from each mouse. Mice were not weaned before experimental use and female vaginal opening had not occurred before experimental use. Pups were ear punched for identification and genotyping. Mice were housed in a temperature- and light-controlled room (22 ± 1°C, 40–45% humidity, 12h light/dark cycle, lights on at 7:00 A.M.). All cages were washed polysulfone bisphenol A free and were filled with bedding manufactured from virgin hardwood chips (Beta Chip, NEPCO) to avoid the endocrine disruptors present in corncob bedding (Markaverich et al., 2002; Mani et al., 2005; Villalon Landeros et al., 2012). Soy protein-free rodent chow (2020X, Teklad) and glass-bottle provided water were available *ad libitum.* All animals in these studies were maintained according to the applicable portions of the Animal Welfare Act and the U.S. Department of Health and Human Services *Guide for the Care and Use of Laboratory Animals*, and the study was approved by the Institutional Animal Care and Use Committee.

### Animal genotyping

Mice genotyping was performed by Celplor using the following primers according to The Jackson Laboratory suggested protocol: transgene forward (forward primer, 12153, 5-CTT CTG AGG CGG AAA GAA CC-3), transgene reverse (reverse primer, 12154, 5-TTT CTG ATT GAG AGC ATT CG-3), PCR product length is 750 bp. The internal control was as follows: internal positive control forward (oIMR7338) CTA GGC CAC AGA ATT GAA AGA TCT, internal positive control reverse (oIMR7339) GTA GGT GGA AAT TCT AGC ATC ATC C, PCR product length is 324 bp. PCR was performed according to the suggested protocol from The Jackson Laboratory: 1 cycle of 94°C for 2 min, 5 cycles of 94°C for 30 s, 60–55°C touchdown ramp for 30 s and 72°C for 30 s, 25 cycles of 94°C for 30 s, 55°C for 30 s and 72°C for 30 s, followed by 1 cycle of 72°C for 5 min.

### Acute brain slice preparation

Brain slices for electrophysiological recordings were prepared following a previously published protocol (Dorris et al., 2014). Briefly, mice were deeply anesthetized with isoflurane gas and killed by decapitation. The brain was then dissected rapidly into ice-cold, oxygenated sucrose artificial cerebellum spinal fluid (s-ACSF) containing the following (in mm): 75 sucrose, 1.25 NaH_2_PO_4_, 3 MgCl_2_, 0.5 CaCl_2_, 2.4 Na pyruvate, 1.3 ascorbic acid from Sigma-Aldrich, and 75 NaCl, 25 NaHCO_3_, 15 dextrose, 2 KCl from Fisher. The osmolarity of the s-ACSF was between 295 and 305 mOsm, and pH was between 7.2 and 7.4. Coronal brain slices (300 µm) were prepared using a vibratome and then incubated in regular ACSF containing the following (in mm): 126 NaCl, 26 NaHCO_3_, 10 dextrose, 3 KCl, 1.25 NaH_2_PO_4_, 1 MgCl_2_, 2 CaCl_2_ (295–305 mOsm, pH 7.2–7.4) for 30 min at 30 ± 1°C, and then at least 30 min at room temperature (21–23°C). Slices were stored submerged in room temperature, oxygenated ACSF for up to 5 h after sectioning in a large volume bath holder.

### Electrophysiological recording

**Figure 1.**
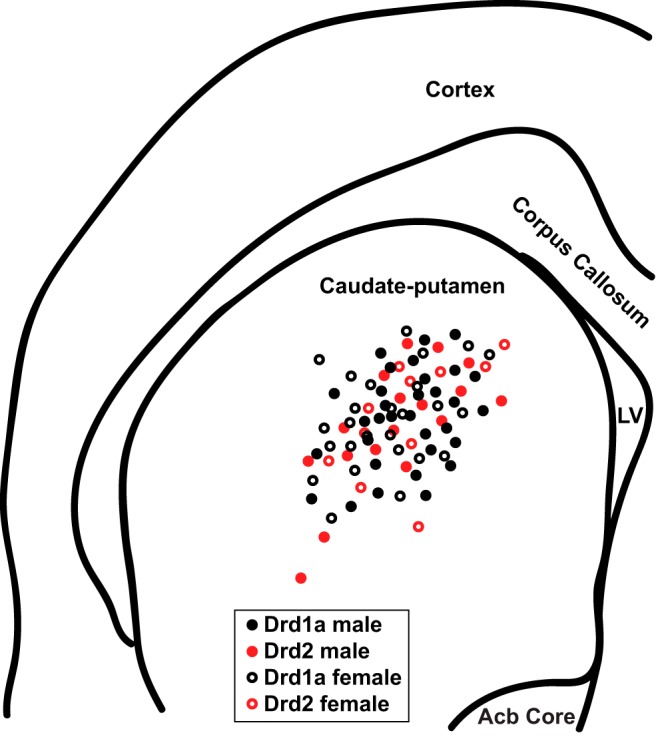
Whole-cell patch-clamped MSN location in the caudate-putamen of female and male Drd1a-tdTomato line 6 BAC transgenic mice. Drd1a males and females represent recordings from fluorescently-labeled Drd1a-positive MSNs. Drd2 males and females represent recordings from non-fluorescently labeled MSNs. LV, Lateral ventricle; AC, anterior commissure; ACB, nucleus accumbens.

Slices were allowed to rest at least 1 h after sectioning, and were then placed in a Zeiss Axioskop equipped with IR-DIC and fluorescent optics, a Dage IR-1000 video camera, and 10× and 40× lenses with optical zoom. Slices were superfused with oxygenated ACSF heated to 28 ± 0.2°C. Whole-cell patch-clamp recordings were used to record the electrical properties of fluorescently labeled Drd1a and unlabeled Drd2 MSNs in the caudate-putamen (Fig. 1). Caudate-putamen gross regional volume and cell density and soma size do not grossly vary by sex in rodents and humans (Meitzen et al., 2011; Wong et al., 2016). Glass patch electrodes contained the following solution (in mm): 115 K d-gluconate, 8 NaCl, 2 EGTA, 2 MgCl_2_, 2 MgATP, 0.3 NaGTP, 10 phosphocreatine from Sigma-Aldrich, and 10 HEPES from Fisher (285 mOsm, pH 7.2–7.4). Signals were amplified, filtered (2 kHz), and digitized (10 kHz) with a MultiClamp 700B amplifier attached to a Digidata 1550 system and a personal computer using pClamp 10 software. Membrane potentials were corrected for a calculated liquid junction potential of −13.5 mV. Using previously described procedures (Dorris et al., 2015), recordings were first made in current-clamp to assess neuronal action potential and passive membrane properties. MSNs were identified by their medium-sized somas, the presence of a slow ramping subthreshold depolarization in response to low-magnitude positive current injections, a hyperpolarized resting membrane potential more negative than −65 mV, inward rectification, and prominent spike after hyperpolarization (O’Donnell and Grace, 1993; Belleau and Warren, 2000). After MSN identification and current-clamp recording, oxygenated ACSF containing both the GABA_A_ receptor antagonist picrotoxin (PTX;150 µm; Fisher) and the voltage-gated sodium channel blocker tetrodotoxin (TTX; 1 µm, Abcam Biochemicals) was applied to the bath solution to abolish GABAergic IPSC events and action potentials, respectively. Following an established protocol (Cao et al., 2016), once depolarizing current injection no longer generated an action potential after exposure to TTX and PTX, MSNs were voltage-clamped at −70 mV and miniature mEPSCs were recorded for at least 5 min. In all experiments input/series resistance was monitored for changes and cells were excluded if resistance changed >25%.

### Data analysis

Intrinsic electrophysiological properties, action potential and mEPSC characteristics were recorded and analyzed using pClamp 10. After break-in, the resting membrane potential was first allowed to stabilize for ∼1–2 min, as in (Mu et al., 2010). Then, at least three series of depolarizing and hyperpolarizing current injections were applied to elicit basic neurophysiological properties. The electrophysiological properties measured followed previously described definitions (Dorris et al., 2015; Cao et al., 2016; Willett et al., 2016, 2018), which were based on those of Perkel and colleagues (Farries and Perkel, 2000, 2002; Farries et al., 2005; Meitzen et al., 2009). For each neuron, measurements were made of at least three action potentials generated from the minimum current injection necessary to elicit one or two action potentials. These measurements were then averaged to generate the reported action potential measurements for that neuron. For action potential measurements, only the first generated action potential was analyzed. Action potential threshold was defined as the first point of sustained positive acceleration of voltage (δ^2^V/δ*t* ^2^) that was also more than 3× SD of membrane noise before the detected threshold (Baufreton et al., 2005). The slope of the linear range of the evoked firing rate to positive current curve (FI slope) was calculated from the first current stimulus that evoked an action potential to the first current stimulus that generated an evoked firing rate that persisted for at least two consecutive current stimuli. Input resistance in the linear, non-rectified range was calculated from the steady-state membrane potential in response to −0.02 nA hyperpolarizing injected current. Rectified range input resistance, inward rectification, and percentage inward rectification (RRIR/IR × 100) was calculated using the most hyperpolarizing current injected into the MSNs, as previously described (Belleau and Warren, 2000). The membrane time constant was calculated by fitting a single exponential curve to the membrane potential change in response to −0.02 nA hyperpolarizing pulses. mEPSC frequency, amplitude, and decay were analyzed off-line using Mini Analysis (Synaptosoft, http://www.synaptosoft.com/MiniAnalysis/). Threshold was set as 5 pA, noise filter was set at 1000 Hz, and accurate event detection was validated by visual inspection.

### Statistics

Experiments were analyzed via a two-way ANOVA with a Tukey’s multiple-comparisons *post hoc* test (Excel v2010; Microsoft; Prism v6.07, GraphPad Software). *p* values < 0.05 were considered a priori as significant. Values 6 SD away from the mean were a priori excluded from analysis. Effect size was assessed using Cohen’s *d* value (Calin-Jageman, 2018). *d* values are reported numerically and were classified a priori as small (>0.20), medium (>0.50), and large (>0.80; Cohen, 1977). Data are presented as mean ± SEM.

## Results

A total of 86 MSNs from the caudate-putamen of male and female B6 *Cg-Tg (Drd1a-*tdTomato) 6 Calak/J hemizygous mice were recorded for this study. Recorded MSNs were a priori sorted into four experimental groups: male tdTomato-labeled Drd1a-positive MSNs, female tdTomato-labeled Drd1a-positive MSNs, male tdTomato-unlabeled MSNs, and female tdTomato-unlabeled MSNs. MSNs unlabeled by tdTomato fluorescence nearly exclusively comprise the Drd2-positive MSN subtype, including during the developmental age and striatal region assessed in this study (Ade et al., 2011; Enoksson et al., 2012; Thibault et al., 2013). tdTomato-unlabeled MSNs have rare (∼1.6%) contamination with Drd1a-positive MSNs. Thus, for convenience in this study we refer to all tdTomato-unlabeled MSNs as Drd2 MSNs, with the full acknowledgment that this designation is putative.

### Action potential properties

**Figure 2.**
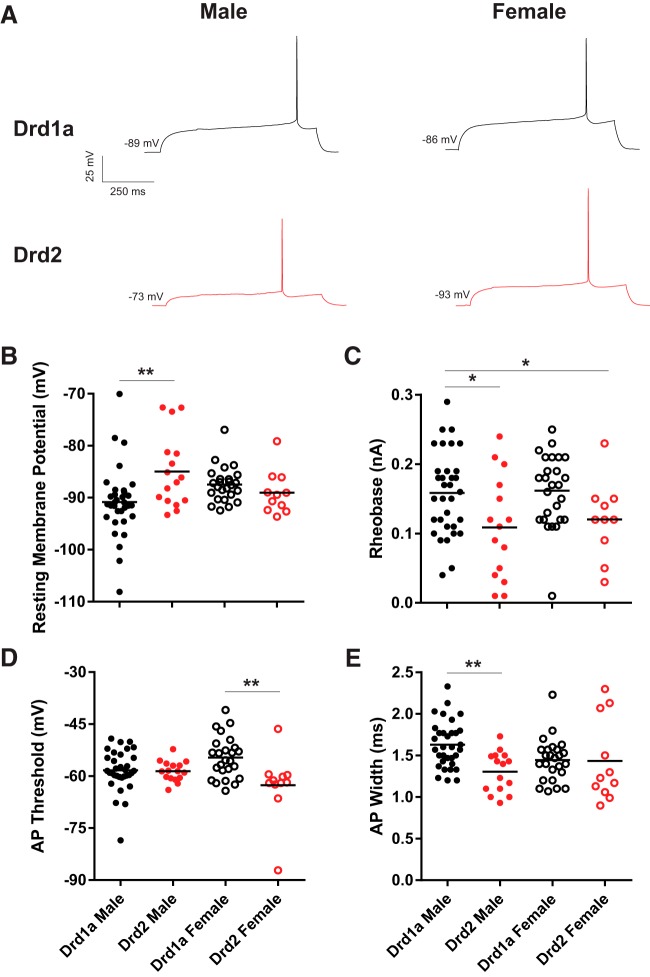
Action potential rheobase, threshold, and width vary by MSN subtype. ***A***, Voltage response of male and female Drd1a and Drd2 MSN subtypes to a depolarizing rheobase current injection. ***B***, Resting membrane potential exhibited greater diversity in male Drd1A MSNs. ***C***, Action potential rheobase is increased in Drd1a MSNs compared with Drd2 MSNs. ***D***, Action potential threshold is depolarized in Drd1a MSNs compared with Drd2 MSNs, and interacts with sex. ***E***, Action potential width is longer in Drd1a MSNs compared with Drd2 MSNs, and interacts with sex. AP, Action potential. *p < 0.05, **p < 0.01.

To test the hypothesis that action potential properties differed across MSN subtype and animal sex, MSNs were current-clamped and injected with increasing amounts of depolarizing current to elicit action potential generation (Fig. 2*A*). The resting membrane potential, rheobase, action potential threshold, width, amplitude, action potential afterhyperpolarization peak amplitude and time to afterhyperpolarization peak amplitude were assessed (Table 2). MSNs exhibited differences between subtypes or interactions between subtype and sex in several attributes, including the resting membrane potential (Fig. 2*B*). Compared between groups, the resting membrane potential of male Drd1a MSNs was hyperpolarized compared with male Drd2 MSNs (*p* < 0.01; *d* = 0.86), but not between female Drd1a MSNs compared with female Drd2 MSNs (*p* > 0.05, *d* = 0.42). Rheobase, or the minimum current sufficient for eliciting action potential generation, was increased in Drd1a MSNs compared with Drd2 MSNs (Fig. 2*C*). Compared between groups, the rheobase of male Drd1a MSNs differed from male and female Drd2 MSNs (*p* < 0.05, *d* = 0.74; *p* < 0.05, *d* = 0.77; respectively). The action potential threshold was hyperpolarized in Drd1a MSNs compared with Drd2 MSNs (Fig. 2*D*). Compared between groups, the action potential threshold of female Drd1a MSNs differed from female Drd2 MSNs (*p* < 0.01, *d* = 1.00), but not between male Drd1a MSNs and male Drd2 MSNs (*p* > 0.05, *d* = 0.05). The action potential width of Drd1a MSNs was longer compared with Drd2 MSNs (Fig. 2*E*). Compared between groups, the action potential width of male Drd1a MSNs was increased compared with male Drd2 MSNs (*p* < 0.01, *d* = 1.24), but not between female Drd1a MSNs and female Drd2 MSNs (*p* > 0.05, *d* = 0.03). Considering other passive properties, no differences were detected between MSN subtype and sex in action potential amplitude, action potential afterhyperpolarization peak amplitude and time to afterhyperpolarization peak amplitude (Table 2). These differences in action potential properties indicate that Drd1a MSNs are less likely to generate an action potential at low magnitudes of injected depolarizing current than are Drd2 MSNs.

**Table 2.** Electrophysiological properties of male and female Drd1a and Drd2 mouse caudate-putamen medium spiny neurons

**Property**	**Drd1a**	**Drd2**	**Statistics (*F*, *p*)**
Resting potential, mV	Male: −90.9 ± 1.2*^a^* Female: −87.5 ± 0.7*^a^* ^,^*^b^*	Male: −85.0 ± 1.8*^b^* Female: −89.0 ± 1.2*^a^* ^,^*^b^*	**Interaction: *F*_(1,82)_ = 8.7; *p* = 0.004** Sex: *F*_(1,82)_=0.1; *p* = 0.75 Subtype: *F*_(1,82)_=2.8; *p* = 0.10 ***post hoc*: Tukey’s**
Rheobase, nA	Male: 0.16 ± 0.01^a^ Female: 0.16 ± 0.01^a^	Male: 0.10 ± 0.02*^b^* Female: 0.12 ± 0.02*^a^* ^,^*^b^*	Interaction: *F*_(1,82)_ = 0.4; *p* = 0.52 Sex: *F*_(1,82)_=0.4; *p* = 0.54 **Subtype:** *F***_(1,82)_=12.3;** *p* **= 0.0007** ***post hoc*: Tukey’s**
AP threshold, mV	Male: −58.3 ± 1.0^a,b^ Female: −54.6 ± 1.2^a^	Male: −58.6 ± 0.7*^a^* ^,^*^b^* Female: −62.6 ± 2.9*^b^*	**Interaction: *F*_(1,81)_=7.2; *p* = 0.0087** Sex: *F*_(1,81)_=0.01; *p* = 0.90 **Subtype: F_(1,81)_=8.0; *p* = 0.0058** ***post hoc*: Tukey’s**
AP amplitude, mV	Male: 68.8 ± 2.1 Female: 69.5 ± 2.9	Male: 69.3 ± 2.8 Female: 79.1 ± 3.1	Interaction: *F*_(1,81)_=2.3; *p* = 0.13 Sex: *F*_(1,81)_=3.1; *p* = 0.08 Subtype: *F*_(1,81)_=3.0; *p* = 0.09
AP width at half-peak, ms	Male: 1.63 ± 0.05^a^ Female: 1.45 ± 0.05^a,b^	Male: 1.31 ± 0.06*^b^* Female: 1.43 ± 0.15^a,b^	**Interaction: *F*_(1,80)_=4.7; *p* = 0.0339** Sex: *F*_(1,80)_=0.1; *p* = 0.7052 **Subtype: *F*_(1,80)_=5.3; *p* = 0.0234** ***post hoc*: Tukey’s**
AHP peak, mV	Male: −8.9 ± 0.4 Female: −11.0 ± 0.7	Male: −9.8 ± 0.8 Female: −9.2 ± 0.7	Interaction: *F*_(1,80)_=3.5; *p* = 0.0654 Sex: *F*_(1,80)_=0.8; *p* = 0.37 Subtype: *F*_(1,80)_=0.2; *p* = 0.69
AHP time to peak, ms	Male: 32.2 ± 1.3 Female: 26.3 ± 1.6	Male: 31.8 ± 3.4 Female: 32.5 ± 3.2	Interaction: *F*_(1,81)_=2.1; *p* = 0.15 Sex: *F*_(1,81)_=1.4; *p* = 0.24 Subtype: *F*_(1,81)_=1.6; *p* = 0.21
FI slope, Hz/nA	Male: 192.5 ± 10.4^a^ Female: 164.0 ± 12.8^a^	Male: 278.7 ± 28.9*^b^* Female: 242.0 ± 23.0*^b^*	Interaction: *F*_(1,81)_=0.1; *p* = 0.82 Sex: *F*_(1,81)_=3.4; *p* = 0.0708 **Subtype: *F*_(1,81)_=21.2; *p* < 0.0001** ***post hoc*: Tukey’s**
Linear range input resistance, MΩ	Male: 102.1 ± 5.8^a^ Female: 93.6 ± 6.6^a^	Male: 141.0 ± 19.2*^b^* Female: 109.8 ± 18.3*^a^* ^,^*^b^*	Interaction: *F*_(1,78)_=1.1; *p* = 0.30 Sex: *F*_(1,78)_=2.9; *p* = 0.09 **Subtype: *F*_(1,78)_=6.3; *p* = 0.0138** ***post hoc*: Tukey’s**
Rectified range input resistance, MΩ	Male: 83.4 ± 4.7 Female: 83.9 ± 8.6	Male: 112.0 ± 17.7 Female: 83.1 ± 11.7	Interaction: *F*_(1,79)_=2.0; *p* = 0.16 Sex: *F*_(1,79)_=1.9; *p* = 0.17 Subtype: *F*_(1,79)_=1.8; *p* = 0.18
Inward rectification, %	Male: 82.3 ± 1.3 Female: 81.0 ± 1.8	Male: 80.8 ± 4.3 Female: 79.2 ± 3.3	Interaction: *F*_(1,79)_=0.0; *p* = 0.93 Sex: *F*_(1,79)_=0.3; *p* = 0.57 Subtype: *F*_(1,79)_=0.4; *p* = 0.52
Time constant of the membrane, ms	Male: 10.3 ± 0.8 Female: 19.6 ± 1.2	Male: 14.6 ± 3.2 Female: 9.9 ± 1.9	Interaction: *F*_(1,82)_=2.9; *p* = 0.09 Sex: *F*_(1,82)_=1.2; *p* = 0.28 Subtype: *F*_(1,82)_=1.6; *p* = 0.20
Capacitance, pF	Male: 109.6 ± 10.0 Female: 99.0 ± 7.0	Male: 94.4 ± 11.2 Female: 91.2 ± 8.5	Interaction: *F*_(1,78)_=0.1; *p* = 0.74 Sex: *F*_(1,78)_=0.4; *p* = 0.55 Subtype: *F*_(1,78)_=1.0; *p* = 0.31
mEPSC frequency, Hz	Male: 1.9 ± 0.2 Female: 1.9 ± 0.2	Male: 2.0 ± 0.5 Female: 1.4 ± 0.4	Interaction: *F*_(1,47)_=0.9; *p* = 0.33 Sex: *F*_(1,47)_=1.2; *p* = 0.29 Subtype: *F*_(1,47)_=0.3; *p* = 0.58
mEPSC amplitude, pA	Male: 15.9 ± 0.4^a^ Female: 15.2 ± 0.6^a^	Male: 17.7 ± 1.1*^a^* ^,^*^b^* Female: 20.7 ± 1.5*^b^*	**Interaction: *F*_(1,47)_=5.2; *p* = 0.0270** Sex: *F*_(1,47)_=2.1; *p* = 0.1498 **Subtype: *F*_(1,47)_=20.4; *p* < 0.0001** ***post hoc*: Tukey’s**
mEPSC decay, ms	Male: 1.9 ± 0.2 Female: 1.9 ± 0.2	Male: 2.0 ± 0.5 Female: 1.4 ± 0.4	Interaction: *F*_(1,47)_=2.6; *p* = 0.11 Sex: *F*_(1,47)_=0.1; *p* = 0.76 Subtype: *F*_(1,47)_=0.0; *p* = 0.95

Values are mean ± SEM. Bold font indicates statistical significance. Different superscript letters denote significant differences detected by a Tukey’s *post hoc* test. AP, action potential; AHP, afterhyperpolarization; FI, frequency of evoked spikes to injected depolarization current.

### Intrinsic excitability and action potential generation rates

**Figure 3.**
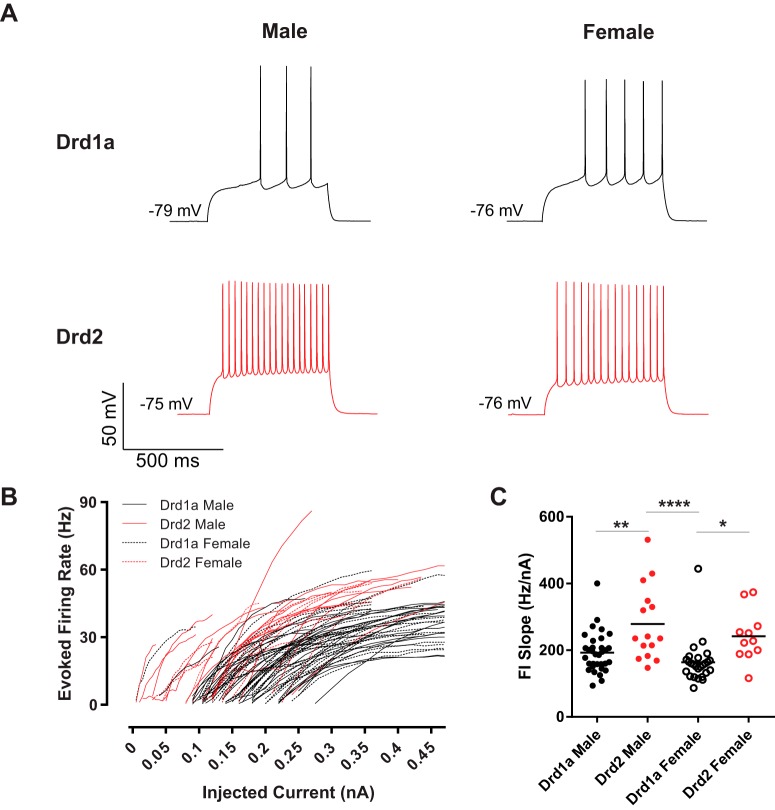
Action potential firing rates evoked by depolarizing current injections vary by MSN subtype. ***A***, Voltage response of male and female Drd1a and Drd2 MSN subtypes to a depolarizing post-rheobase current injection. ***B***, Drd1a MSNs exhibited decreased action potential firing rates evoked by depolarizing current injections compared with Drd2 MSNs. ***C***, The slope of the evoked action potential to depolarizing current injection curve (FI slope) differed by MSN subtype, with Drd2 MSNs exhibiting increased excitability compared with Drd1a MSNs. FI slope, Slope of the evoked action potential to depolarizing current injection curve. *p < 0.05, **p < 0.01, ****p < 0.0001.

These differences between MSN subtype rheobase, action potential threshold, width, and time to first action potential properties indicate that overall MSN excitability may also differ by subtype (Fig. 3*A*). To assess this, we began by analyzing the frequency of action potentials evoked by depolarizing current injections. Action potential firing rates evoked by depolarizing current injections were visibly decreased in Drd1a compared with Drd2 MSNs in both males and females (Fig. 3*B*). To further probe the relationship between MSN subtype and action potential generation, we quantified the slope of the evoked firing rate to positive current curve (FI slope). FI slope differed by subtype but not sex, with Drd1a MSNs exhibiting decreased excitability compared with Drd2 MSNs (Fig. 3*C*). Compared between groups, the FI slope of male Drd1a MSNs differed from male Drd2 MSNs (*p* < 0.01, *d* = 0.96). Female Drd1A MSNs differed from male Drd2 MSNs (*p* < 0.0001, *d* = 1.25) and female Drd2 MSNs (*p* < 0.05, *d* = 1.10). These data indicate that excitability robustly differs between MSN subtypes.

### Passive membrane properties

**Figure 4.**
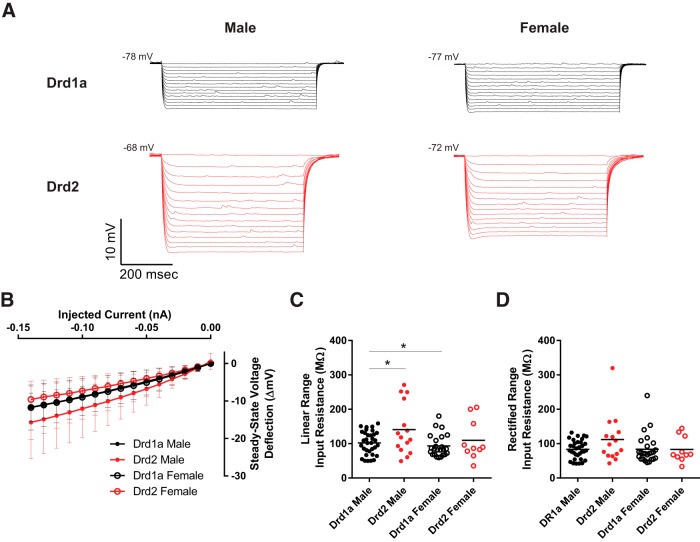
Input resistance varies by MSN subtype. ***A***, Voltage response of male and female Drd1a and Drd2 MSN subtypes to a series of increasingly negative current injections (−0.01 nA current steps). ***B***, Injected negative current to steady-stage voltage deflection curve (*I–V* curve). Legend, Red solid circles with red line, Drd1a males; black solid circles with black line, Drd2 males; red open circles with red line, Drd1a females; black open circles with black line, Drd2 females. ***C***, Input resistance in the linear range is moderately decreased in Drd1a MSNs compared with Drd2 MSNs. ***D***, Input resistance in the rectified range does not differ between subtypes. *p < 0.05.

To test the hypothesis that MSN passive electrophysiological properties differed by subtype and sex, a series of increasingly negative current pulses were injected into individual neurons (Fig. 4*A*). MSN subtypes exhibited differences in input resistance across both the linear and rectified ranges (Fig. 4*B*). Input resistance in the linear and rectified ranges, percentage inward rectification, time constant of the membrane, and capacitance were analyzed (Table 2). Linear range input resistance was largely decreased in Drd1a MSNs compared with Drd2 MSNs (Fig. 4*C*). Compared between groups, the linear range input resistance of male Drd1a MSNs differed from male Drd2 MSNs (*p* < 0.05. *d* = 0.67), but did not differ between female Drd1a MSNs and female Drd2 MSNs (*p* > 0.05, *d* = 0.34). Male Drd2 MSNs also differed from female Drd1a MSNs (*p* < 0.05, *d* = 0.83). Rectified range input resistance did not differ between MSN subtypes (Fig. 4*D*) or other measures of inward rectification (Table 2). Considering other passive properties, no differences were detected between MSN subtype or sex in the time constant of the membrane, and capacitance (Table 2). Collectively, these analyses indicate that input resistance varies between MSN subtypes, with no differences in other passive membrane properties.

### mEPSC properties

**Figure 5.**
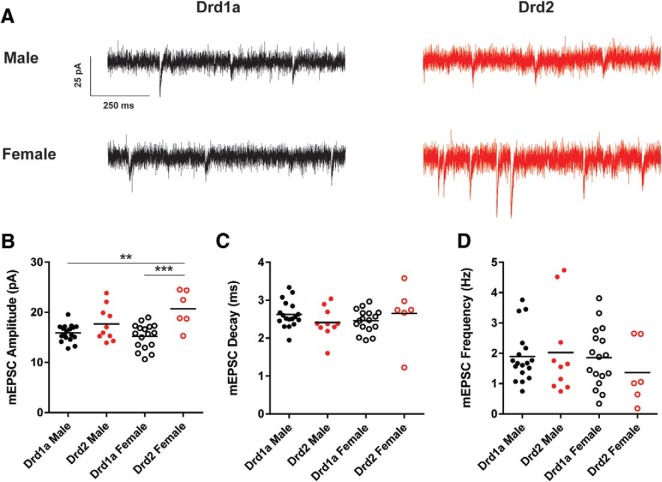
mEPSC properties vary by MSN subtype. ***A***, mEPSCs recorded from male and female Drd1a and Drd2 MSN subtypes. MSNs were voltage-clamped at −70 mV and mEPSCs were recorded in the presence of TTX and PTX to block voltage-gated sodium channels and GABAergic synaptic activity, respectively. ***B***, mEPSC amplitude was increased in Drd1a MSNs compared with Drd2 MSNs. ***C***, mEPSC decay did not differ by subtype or sex. ***D***, mEPSC frequency did not differ by subtype or sex. **p < 0.01, ***p < 0.001.

We voltage-clamped 18 male and 17 female Drd1a MSNs and 10 male and 6 female Drd2 MSNs at −70 mV and recorded mEPSCs in the presence of TTX and PTX (Fig. 5*A*). mEPSC frequency, amplitude and decay were analyzed (Table 2). mEPSC amplitude was increased in Drd1a MSNs compared with Drd2 MSNs (Fig. 5*B*). Compared between groups, the mEPSC amplitude of male Drd1a MSNs differed from female Drd2 MSNs (*p* < 0.01, *d* = 0.82) but not male Drd2 MSNs (*p* > 0.05, *d* = 0.67). The mEPSC amplitude of female Drd1a MSNs differed from female Drd2 MSNs (*p* < 0.001, *d* = 1.74). mEPSC decay did not differ between MSN subtype or sex (Fig. 5*C*). Likewise, mEPSC frequency did not differ between MSN subtype or sex (Fig. 5*D*). These data indicate that mEPSC amplitude differs between MSN subtypes.

## Discussion

MSNs form at least two major pathways depending on their dopaminergic receptor and neuropeptide expression, electrophysiological properties, where they project, and ultimately their effect on behavior. The caudate-putamen MSNs of the direct pathway predominantly express D1-dopamine receptors, contain substance P and dynorphin, project to the basal ganglia output nuclei, and stimulate downstream behavioral output. Indirect pathway MSNs express D2-dopamine receptors, contain encephalin, project to the lateral globus pallidus, and lead to inhibition of downstream behavioral output. This study comprehensively evaluates mouse caudate-putamen MSN subtype electrophysiological properties, extending previous studies that targeted a smaller battery of electrical properties and that were performed solely in males or mice of unknown sex (Table 1). Electrophysiological properties differed between MSN subtypes, with Drd2 MSNs exhibiting increased intrinsic excitability compared with Drd1a MSNs, which is indicated most notably by differences in rheobase, action potential threshold, input resistance in the linear range, and increased FI slope. Interestingly, this robust set of properties exhibits varying degrees of consistence with previous literature on MSN subtype electrophysiology, and select electrophysiological properties showed statistical interactions between subtype and biological sex.

The detected increase in excitability in Drd2 MSNs is generally consistent with previous studies of MSN subtypes in rodents across striatal regions, although there are subtle differences depending on the assessed electrophysiological metric and perhaps age (Onn et al., 1994a, b,c; Venance and Glowinski, 2003; Kreitzer and Malenka, 2007; Ade et al., 2008; Cepeda et al., 2008; Gertler et al., 2008; Chan et al., 2012; Ma et al., 2012; Planert et al., 2013; Reig and Silberberg, 2014; Maurice et al., 2015; Cao et al., 2018; Goodliffe et al., 2018). Perhaps the most consistent metric indicating increased excitability in Drd2 MSNs compared with Drd1a MSNs is the decreased rheobase in Drd2 MSNs. Every study that has assessed this property has detected this difference, despite using varying protocols and electrophysiological methods. From an electrophysiological perspective, differences in rheobase are rarely the sole electrophysiological difference between neuron types. Generally, a shift in rheobase is accompanied by concomitant changes in properties such as resting membrane potential, input resistance in the linear range, and/or action potential threshold. For instance, Gertler et al. (2008) detected changes in rheobase accompanied by changes in resting membrane potential and input resistance, but not action potential threshold. Planert et al. (2013) detected changes in rheobase accompanied by a change in input resistance, but not action potential threshold or resting membrane potential in rats but not mice. Cepeda et al. (2008) did not assess rheobase, but did detect a difference in action potential threshold. The current study detected a difference in rheobase accompanied by changes in action potential threshold and input resistance, supporting a model where the decreased rheobase values in Drd2 MSN subtypes is largely driven by a hyperpolarized action potential threshold and an increased input resistance. The increase in excitability observed in Drd2 MSNs could ultimately translate to a decrease in behavioral output. However, this particular interpretation is highly tentative given that MSNs make complex calculations between dopamine, glutamate, intrinsic properties, and other neuromodulators. Interestingly, we detected an interaction between MSN subtype and sex in resting membrane potential and action potential threshold. Thus, it is possible that the reason why the results of our study differ from those of Gertler et al. (2008) is because of the use of animals of undetermined sex in that study.

Regarding excitatory synaptic input, the current study detected increased mEPSC amplitude in Drd2 MSN subtypes compared with Drd1a MSN subtypes. To our knowledge, this is the first indication that mEPSC amplitude can differ by MSN subtype (Table 1). This may be because of a variance and power interaction, although previous studies of MSN subtypes used similar experimental *N*. This finding does align with previous research which detected large amplitude AMPA-mediated synaptic events in Drd2 MSNs that were not seen in Drd1a MSNs (Cepeda et al., 2008). The recording conditions under which the mEPSCs were assessed in this study eliminate non-AMPA-mediated currents (Proaño et al., 2018). A number of factors could potentially mediate this difference in mEPSC amplitude, including morphologic differences and/or differences in AMPA receptor number or subunit composition (Tallaksen-Greene and Albin, 1996; Vorobjev et al., 2000). Supporting this, there is evidence that the size of corticostriatal presynaptic terminals is larger on Drd2 MSN spines compared with Drd1a MSN spines (Lei et al., 2004). One possibility why previous studies did not detect a significant difference in mEPSC amplitude is because the effect size for this particular attribute is larger in females compared with males. Previous studies either only tested males or did not report sex, or sex-specific findings. Following this, two previous studies detected a greater sEPSC and mEPSC frequency in caudate-putamen Drd2 MSNs compared with Drd1a MSNs that is not accompanied by differences in amplitude or decay (Kreitzer and Malenka, 2007; Cepeda et al., 2008). However, this literature is mixed because Cepeda et al. (2008) detected a difference in mEPSC rise time, Day et al. (2006) did not detect a difference in mEPSC frequency in prepubertal animals, and Goodliffe et al. (2018) did not detect a difference in mEPSC frequency in adult animals. It is possible that this variability in findings is in some part explained by the neglect or overrepresentation of one sex compared with another.

There are other factors which may also play a role, including animal age. Most studies of MSN subtype properties used mice that were between P17 and P30 (Table 1). During these periods, various levels of MSN synaptic maturation are occurring, which could contribute to variance in mEPSC properties (Tepper et al., 1998; Uryu et al., 1999). Gertler et al. (2008) used a wide variety of ages to demonstrate that MSN subtype intrinsic properties differ before puberty, but the study did not assess excitatory synaptic properties. Goodliffe et al. (2018) assessed at a much older age (∼P365), and found differences in intrinsic properties but not mEPSC properties (Table 1). Thus, our assessment of the literature is that there is ample evidence for differences in MSN subtype electrophysiological properties prepuberty, but that there is a real need for further studies in adult animals, especially within the context of excitatory synapse properties, sex-specific hormone dynamics, and animal sex, especially because the current study is the only available analysis by sex in mouse caudate-putamen.

Similarly, Drd1a and Drd2 MSN subtypes display different sensitivities to neuromodulators such as dopamine during prepubertal development (Lieberman et al., 2018). Indeed, it has been documented in multiple striatal regions that dopamine receptor expression and/or action shows sex-specific effects during puberty (Andersen et al., 1997, 2002; Kopec et al., 2018). Interestingly, excitatory currents generated by pyramidal tract stimulation show an increased amplitude in Drd1a MSNs compared with Drd2 MSNs in adult male and female mice that were not analyzed with regard to sex (Kress et al., 2013). In *in vivo* experiments in adult female mice, Drd1a MSNs were found to be more responsive to excitatory glutamatergic input compared with Drd2 MSNs (Escande et al., 2016). However, this study used the line 5 Drd1a-tdTomato BAC-transgenic mice, which express properties such as an X-linked inheritance pattern and undefined mammary glands that reduces this strain’s utility for assessing interactions between MSN subtypes and sex (Shuen et al., 2008; Ade et al., 2011). Subtype-specific differences in the development of glutamatergic and dopaminergic inputs onto MSNs in the caudate-putamen require further research, especially in the context of biological sex, environmental stimuli, and animals beyond mice.

The strain and/or species is also a relevant factor in explaining differences in MSN subtype electrophysiological properties across studies. For instance, multiple strains of transgenic mice have been used across studies which have used a variety of means to determine MSN subtype identity. Cepeda et al. (2008), Gertler et al. (2008), and the current study all targeted Drd1a or Drd2 expression to identify MSN subtypes (albeit with different transgenic strategies), whereas other studies have used different targets such as the muscarinic M4 receptor locus which labels striatonigral MSNs (Kreitzer and Malenka, 2007). There is some evidence that there is incomplete overlap between M4 and Drd1a dopamine receptors which may contribute to variance in detected electrophysiological properties, including sEPSC frequency between M4 and D1 cells (Bernard et al., 1992; Cepeda et al., 2008). Here we used the B6 *Cg-Tg (Drd1a-*tdTomato) 6 Calak/J hemizygous mice (line 6). We chose this strain because of its established high specificity, in that tdTomato-labeled MSNs almost exclusively consist of Drd1a MSNs, and that tdTomato-unlabeled MSNs exhibit only ∼1.6% contamination with Drd1a MSNs (Ade et al., 2011; Enoksson et al., 2012; Thibault et al., 2013). Furthermore, the tdTomato label is easily detected using standard fluorescent microscopy, optimizing differentiation for whole-cell patch-clamp. Although the line 6 version of this transgenic mouse line does not show the obvious confounds for sex research that the line 5 version displays, including the X-linked inheritance pattern and undefined mammary glands, caution is always necessary with any transgenic mouse line that targets dopamine receptors. It is possible that transgenes targeting dopamine receptors subtly disrupt sexual differentiation, especially given the long documented and recently reaffirmed sex differences and hormone-sensitivity of the dopaminergic system in both rats and mice (Di Paolo, 1994; Becker, 1999; Calipari et al., 2017).

Independent of sex, other mouse lines with transgenic manipulations of the dopamine system by attaching fluorophores have shown aberrant striatal-mediated behaviors, especially when strain and genetic homozygosity were not carefully monitored (Kramer et al., 2011; Chan et al., 2012). We raise this possibility neither to argue that transgenic mice in general are not useful for neuroscience research nor for understanding the effects of sex and steroid sex hormones. Several mouse models have made critical contributions to our understanding of sexual differentiation, most notably the four core genotypes (De Vries et al., 2002), including as applied to the caudate-putamen (Chen et al., 2009). Rather, we argue that the specific disadvantages and advantages of each research animal should be thoughtfully considered, especially for studies of the impact of natural variables such as sex on individual neuron function. There is no “one size fits all” mouse strain, just as there is no “one size fits all” strain of rat or any other research animal, echoing arguments presented by many investigators in diverse contexts (Beach, 1950; Krebs, 1975; Young et al., 2013; Brenowitz and Zakon, 2015; Ellenbroek and Youn, 2016; Klinck et al., 2017; Remage-Healey et al., 2017). Regarding species differences, to our knowledge, there is only one study that has assessed MSN subtype electrophysiological properties in the caudate-putamen of a species other than mice. Similar to the current study in mice, Planert et al. (2013) found a difference in rheobase and related properties in prepubertal rats of unreported sex. Synaptic properties were not assessed.

Further complicating interpretation was the number of interactions between MSN subtype and sex detected by the current study. Given that animals were assessed before pubertal onset but after the perinatal critical period for hormone-induced organization of the neural substrate, it is possible that these sex differences were generated through some combination of masculinizing/defeminizing hormone action, genes, or epigenetics. All three of these mechanisms are potentially at work in the caudate-putamen and could contribute to the MSN subtype and sex interactions observed here (Chen et al., 2009; Cao et al., 2016). Previous studies in mice used only males, animals of unreported sex, or animals of both sex sexes that were pooled for data analysis. This lack of consideration of biological sex is problematic given the long-known sex differences in striatal mediated behaviors, disorders, MSN properties, and neuromodulator systems such as dopamine (Mermelstein et al., 1996; McLean and Anderson, 2009; Carroll and Anker, 2010; Young and Korszun, 2010; Becker and Chartoff, 2018; Meitzen et al., 2018). Although this work has predominantly been performed in rats, importantly, adult mice exhibit sex differences in striatal gene expression and function in both the caudate-putamen and nucleus accumbens (Chen et al., 2009; Calipari et al., 2017). The current study detected an interaction in action potential threshold, which was also found to differ by sex in prepubertal rats (Dorris et al., 2015). Other electrophysiological properties also differed by sex in rats, including the frequency of evoked action potentials to injected current and the action potential afterhyperpolarization, but were not found to differ by the current study in mice. This difference between mice and rats may be because of a number of potential factors, including but not limited to variance between inbred and outbred rodent strains, overall species differences and the effects of domestication, MSN subtype sampling bias, developmental trajectory, environmental factors such as stress, and/or location within the caudate-putamen or striatum as a whole. For a phylogenetically ancient and highly conserved brain region such as the caudate-putamen, it will be particularly interesting to investigate the intersecting roles of subtype, development, and biological sex in influencing MSN electrophysiological properties across a wide range of animals with divergent reproductive behaviors.

## Synthesis

Reviewing Editor: Cheryl Sisk, Michigan State University Decisions are customarily a result of the Reviewing Editor and the peer reviewers coming together and discussing their recommendations until a consensus is reached. When revisions are invited, a fact-based synthesis statement explaining their decision and outlining what is needed to prepare a revision will be listed below. The following reviewer(s) agreed to reveal their identity: Robert Meisel.

The main goal of this experiment was to provide a comprehensive analysis of the electrophysiological properties of striatal medium spiny neurons in male and female mice. The experimental results are extremely valuable given the within animal analyses of dopamine D1 and D2 receptive medium spiny neurons, along with an analysis of sex differences where they occur. Overall, the design of this descriptive study and the methodology are sound, and the analyses largely seem appropriate for the research question. The methods and results are clearly presented. The novelty of the study is limited, but the results do provide a more comprehensive resource than those previously available for researchers using this transgenic mouse line to study striatal connectivity and function. Both reviewers recommend changes to the manuscript to clarify some of the results and to highlight the usefulness of this contribution.

Statistical Concerns

For three results (Rheobase, FI Slope, and Linear Range Input Resistance) only a main effect of subtype is found in the ANOVA, with no interaction. In this situation it can be concluded that D1R and D2R-expressing MSNs differ from each other regardless of sex. However, each of these measures is still followed up with post hoc tests in the paper. These unnecessary group comparisons should be removed.

Major comments

1. Table 1 compares D1R vs D2R-expressing MSN properties multiple studies including the present manuscript. For clarity, the ‘No’ entry in certain cells should be expanded, since it is not immediately clear what ‘No’ means. This should be clarified by changing the entry to ‘No Change’ or ‘D1 = D2’. A column to indicate how D1R vs D2R-expressing neurons were identified in the referenced studies should be added to aid in comparability between results.

2. Table 2 shows the values of electrophysiological properties measured and the statistical analyses used in the manuscript. The post-hoc tests used should be added to this table to clarify when post hoc tests were applied and which of the two tests described in the Methods were used in each case.

3. The discussion is limited and primarily addresses potential causes of differences between findings in the current manuscript and those of the works cited in Table 1. The discussion is clear, scholarly and thorough, yet at the same time, it does not provide any resolution to differences among studies. The discussion should be expanded to highlight:

(a) functional implications of the current findings. The abstract and introduction frame this paper as a study of differences between striatonigral (D1R-expressing) and striatopallidal (D2R-expressing) MSNs, and therefore the discussion should be revised to extend the results of this descriptive study to provide some insight into the functional meaning of the findings for these two pathways.

(b) How the results of this manuscript may connect to other studies of striatal connectivity and function, especially with regards to the ages chosen for this study and the body of work on striatally mediated behaviors in adult animals. This is discussed only briefly, but should be revised to indicate the importance and usefulness of the current findings.

(c) Strengths and caveats of using this specific transgenic mouse line.

(d) What factors potentially account for observed sex-by-subtype interactions.

Minor Comments

1. The use of ‘prepubertal‘ in the title may lead readers to expect this would be a variable in the experiment. It's fine that it is not, though it's not clear that prepubertal is an appropriate description of the animals in the study. The animals were certainly prepubertal, but so are neonates, but we do not know that this is an important factor. Describing them as weanling, or pre-weanling, seems a more accurate label for their age.

2. As is, Figure 1 does not add much to the manuscript. Reading the methods, one reviewer expected Figure 1 to show a photo of a labeled and unlabeled medium spiny neuron being patched. Perhaps a combination figure of the photos of the patched neurons along with the distribution of the neurons would be a good compromise.

3. It would be helpful to know how many recordings came from individual animals on average in addition to the total number of neurons recorded.

4. On page 9, a set of neurons is described as ‘Drd1a-negative’. Why this designation was used instead of Drd2?

5. In this mouse line, only D1R-expressing neurons are labeled. Unlabeled neurons are putatively considered to be D2R-expressing. This issue is clarified early in the results section by indicating that unlabeled MSNs will be referred to as ‘Drd2 MSNs’ for convenience. For clarity, the designation of ‘Drd2-positive MSNs’ in the discussion (Page 11, line 276) should be changed to simply ‘Drd2 MSNs’ to be in line with the rest of the manuscript.

## Author Response

We appreciate the positive and constructive comments from the reviewers. After careful consideration of the reviews, we have addressed the comments either in this letter or by revising the manuscript. Changes in the manuscript text are indicated with yellow highlighting. Below we provide a point-by-point description of how we addressed each comment. The explanations below are labeled or numbered to directly correspond to the reviewers' comments. Thank you for considering this resubmitted manuscript for publication in eNeuro.

Statistical Concerns

After careful consideration of this comment, we have chosen to retain the post-hoc comparison in these three cases. The reason why, is that the assumption that D1R and D2R-expressing MSN differ from each other regardless of sex is not always correct. For example, regarding Rheobase, Female D2R-expressing MSN do not significantly differ from Male or Female D1R, while Male D2R-expressing MSNs do. Accurately describing the complexity of these differences thus warrants the inclusion of these post-hoc tests. To better document the post-hoc comparisons, we now include all relevant measures in Table 2, so that the reader can easily evaluate the statistical comparison.

Major Comments

1. Revised as requested. We agree that the ‘No’ entry is unclear and we have changed all ‘No’ entries to ‘D1=D2’ (Table 1).

2. Revised as requested (Table 2). Only 1 type of post-hoc test was used and the statistical methods have been edited to fix this error (lines 182-183).

3a. Revised as requested (lines 267-273; 304-307).

3b. Revised as requested (lines 284-285, 335-336, 340-346).

3c. Revised as requested (lines 371-397).

3d. Revised as requested (lines 403-408).

Minor comments

1. After thoughtful consideration we have decided to retain the prepubertal label to remain consistent across our series of studies documenting the role of sex across development in multiple striatal regions and species (Willett, et al., 2018; Cao et al., 2018; Proano et al., 2018; Dorris et al., 2015). These papers have all used either ‘prepubertal’ or ‘adult’ to describe the developmental stage. The exact age and weaning status is specified in the methods (Lines 86-91).

2. Given that the caudate-putamen is a large brain are that exhibits numerous subregional differences, we have retained the map documenting the location of recorded neurons.

3. Revised as requested. We agree with the reviewers and now provide the number of individual animals used for each experimental group as well as the average number of neurons recorded from each mouse (lines 88-90).

4. This designation should be Drd2 and has been changed (line 261).

5. We agree with the reviewers and have changed all designations of ‘Drd2-positive MSNs’ to ‘Drd2 MSNs’ (lines 277, 283, 289-290, 303, 352).
